# Supplementary material for: Elevated Expression of JMJD5 Protein Due to Decreased miR-3656 Levels Contributes to Cancer Stem Cell-Like Phenotypes under Overexpression of Cancer Upregulated Gene 2
Source: Biomolecules. 2022 Jan 12;12(1):122. doi: 10.3390/biom12010122 (PMC8774111; doi:10.3390/biom12010122)
Supplement: Supplementary file 1 [file biomolecules-12-00122-s001.zip › biomolecules-1531123-supplementary.pdf]

Elevated expression of JMJD5 protein due to decreased miR-3656 levels contributes to cancer stem cell-like phenotypes under overexpression of cancer upregulated gene (CUG)2

Natpaphan Yawut[1#], Il-Rae Cho[1#], Phatcharaphon Budluang[1], Sirichat Kaowinn[2], Chutima Kaewpiboon[3], Byeoleun Jeon[4], Sang-Woo Kim[4], Ho Young Kang[5], Min Kyung Kang[6], Sang Seok Koh[6], and Young-Hwa Chung[1*]

[1]BK21 plus, Department of Cogno-Mechatronics Engineering, Optomechatronics Research Center, [4]Department of Biological Science, [5]Department of Microbiology, Pusan National University, Busan 46241, Republic of Korea

[2]Department of General Science and Liberal Arts, King Mongkut's Institute of Technology, Ladkrabang Prince of Chumphon Campus, Chumphon 86160, Thailand

[3]Department of Biology, Faculty of Science, Thaksin University, Patthalung 93210, Thailand

[6]Department of Biomedical Sciences, Dong-A University, Busan 49315, Republic of Korea

Keywords: CUG2, JMJD5, cancer stem cell, KDM8, miR-3656

#; equally contributed

* Corresponding author; Young-Hwa Chung, PhD, Department of Cogno-Mechatronics Engineering, Optomechatronics Research Center, Pusan National University, Busan 46241, Republic of Korea, *Email* ; younghc@pusan.ac.kr

Supplementary Figure S1

(A)

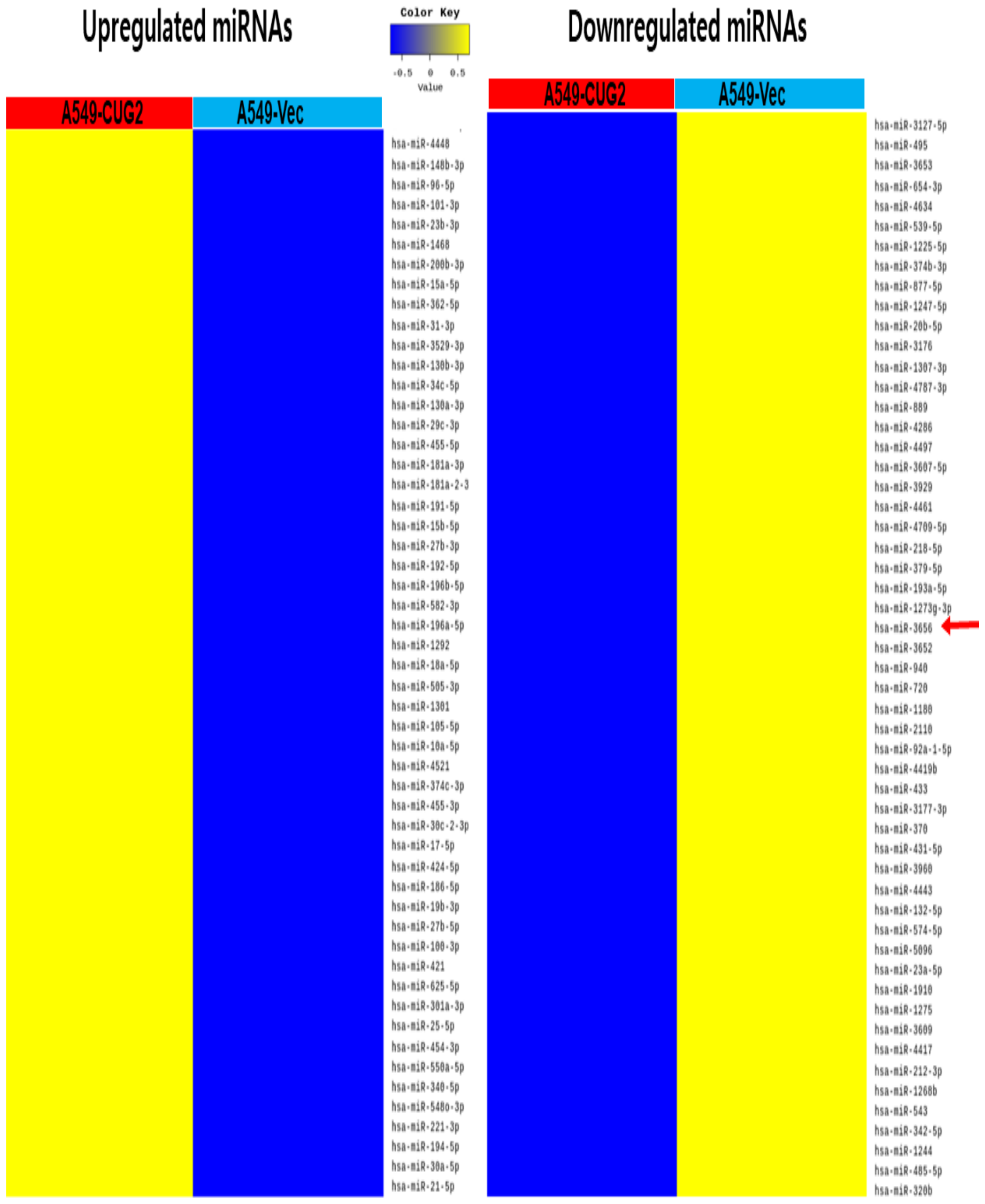

(B)

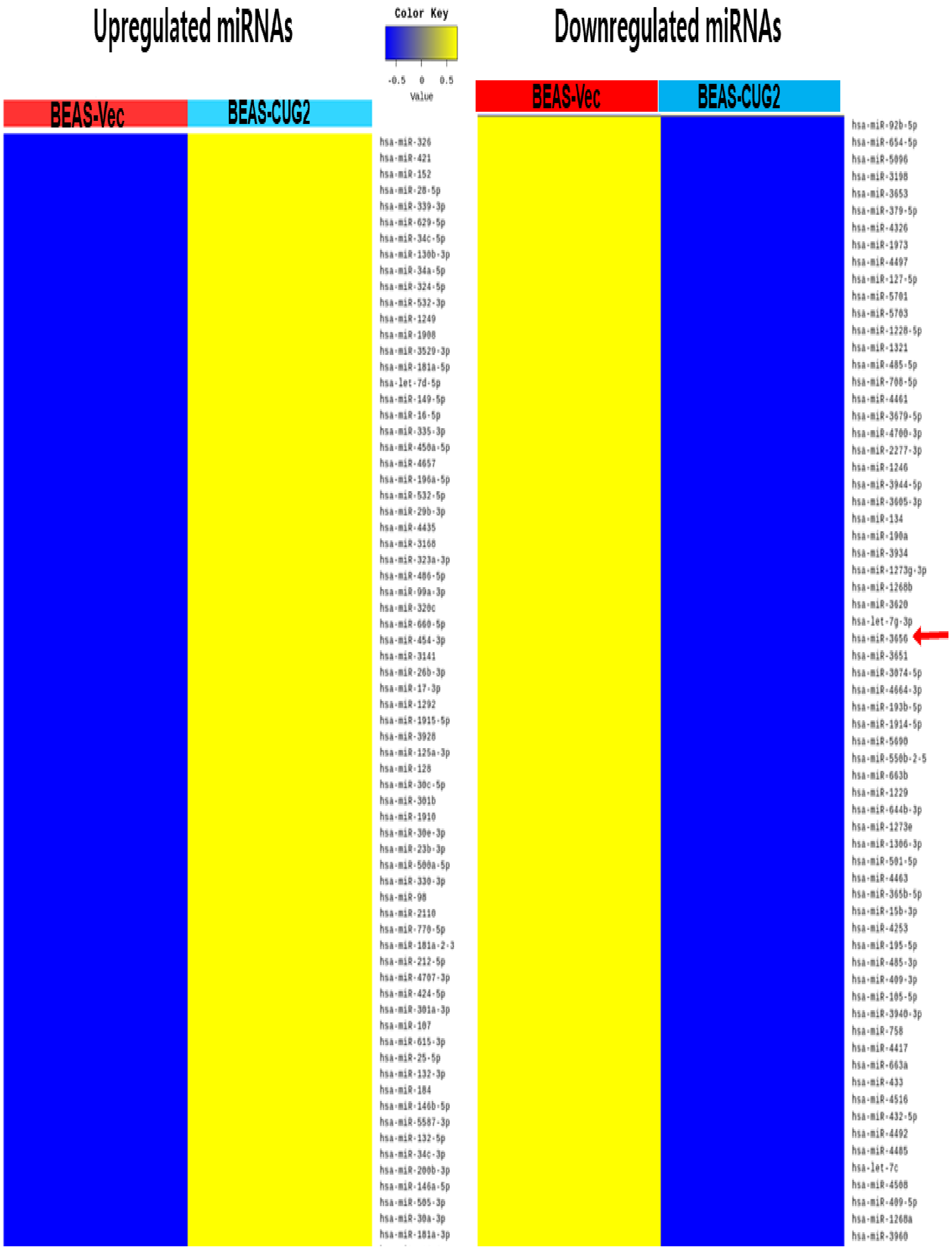

Upregulated miRNAs
Color Key
-0.5 0 0.5
Value
Downregulated miRNAs
BEAS-Vec
BEAS-CUG2
BEAS-Vec
BEAS-CUG2
hsa-miR-326
hsa-miR-421
hsa-miR-152
hsa-miR-28-5p
hsa-miR-339-3p
hsa-miR-629-5p
hsa-miR-34c-5p
hsa-miR-130b-3p
hsa-miR-34a-5p
hsa-miR-324-5p
hsa-miR-532-3p
hsa-miR-1249
hsa-miR-1908
hsa-miR-3529-3p
hsa-miR-181a-5p
hsa-let-7d-5p
hsa-miR-149-5p
hsa-miR-16-5p
hsa-miR-335-3p
hsa-miR-450a-5p
hsa-miR-4657
hsa-miR-196a-5p
hsa-miR-532-5p
hsa-miR-29b-3p
hsa-miR-4435
hsa-miR-3168
hsa-miR-323a-3p
hsa-miR-486-5p
hsa-miR-99a-3p
hsa-miR-320c
hsa-miR-660-5p
hsa-miR-454-3p
hsa-miR-3141
hsa-miR-26b-3p
hsa-miR-17-3p
hsa-miR-1292
hsa-miR-1915-5p
hsa-miR-3928
hsa-miR-125a-3p
hsa-miR-128
hsa-miR-30c-5p
hsa-miR-301b
hsa-miR-1910
hsa-miR-30e-3p
hsa-miR-23b-3p
hsa-miR-500a-5p
hsa-miR-330-3p
hsa-miR-98
hsa-miR-2110
hsa-miR-770-5p
hsa-miR-181a-2-3
hsa-miR-212-5p
hsa-miR-4707-3p
hsa-miR-424-5p
hsa-miR-301a-3p
hsa-miR-107
hsa-miR-615-3p
hsa-miR-25-5p
hsa-miR-132-3p
hsa-miR-184
hsa-miR-146b-5p
hsa-miR-5587-3p
hsa-miR-132-5p
hsa-miR-34c-3p
hsa-miR-200b-3p
hsa-miR-146a-5p
hsa-miR-505-3p
hsa-miR-30a-3p
hsa-miR-181a-3p
hsa-miR-92b-5p
hsa-miR-654-5p
hsa-miR-5096
hsa-miR-3198
hsa-miR-3653
hsa-miR-379-5p
hsa-miR-4326
hsa-miR-1973
hsa-miR-4497
hsa-miR-127-5p
hsa-miR-5701
hsa-miR-5703
hsa-miR-1228-5p
hsa-miR-1321
hsa-miR-485-5p
hsa-miR-708-5p
hsa-miR-4461
hsa-miR-3679-5p
hsa-miR-4700-3p
hsa-miR-2277-3p
hsa-miR-1246
hsa-miR-3944-5p
hsa-miR-3605-3p
hsa-miR-134
hsa-miR-190a
hsa-miR-3934
hsa-miR-1273g-3p
hsa-miR-1268b
hsa-miR-3620
hsa-let-7g-3p
hsa-miR-3656
hsa-miR-3651
hsa-miR-3074-5p
hsa-miR-4664-3p
hsa-miR-193b-5p
hsa-miR-1914-5p
hsa-miR-5690
hsa-miR-550b-2-5
hsa-miR-663b
hsa-miR-1229
hsa-miR-644b-3p
hsa-miR-1273e
hsa-miR-1306-3p
hsa-miR-501-5p
hsa-miR-4463
hsa-miR-365b-5p
hsa-miR-15b-3p
hsa-miR-4253
hsa-miR-195-5p
hsa-miR-485-3p
hsa-miR-409-3p
hsa-miR-105-5p
hsa-miR-3940-3p
hsa-miR-758
hsa-miR-4417
hsa-miR-663a
hsa-miR-433
hsa-miR-4516
hsa-miR-432-5p
hsa-miR-4492
hsa-miR-4485
hsa-let-7c
hsa-miR-4508
hsa-miR-409-5p
hsa-miR-1268a
hsa-miR-3960

Relative expression of miRNA isolated from A549- CUG2 and BEAS-CUG2 cells, and their control cells was measured using a human miRNA microarray (AffymetrixGeneChip® miRNA 4.0 Array, Affymetrix, Santa Clara, USA), which was performed by Macrogen (Seoul, Korea). The Affymetrix Gene Chip miRNA array process was performed according to the manufacturer's protocol. Heat maps of the one-way hierarchical clustering were drafted using Z-score for normalized value (log2 based).

Supplementary Figure S2

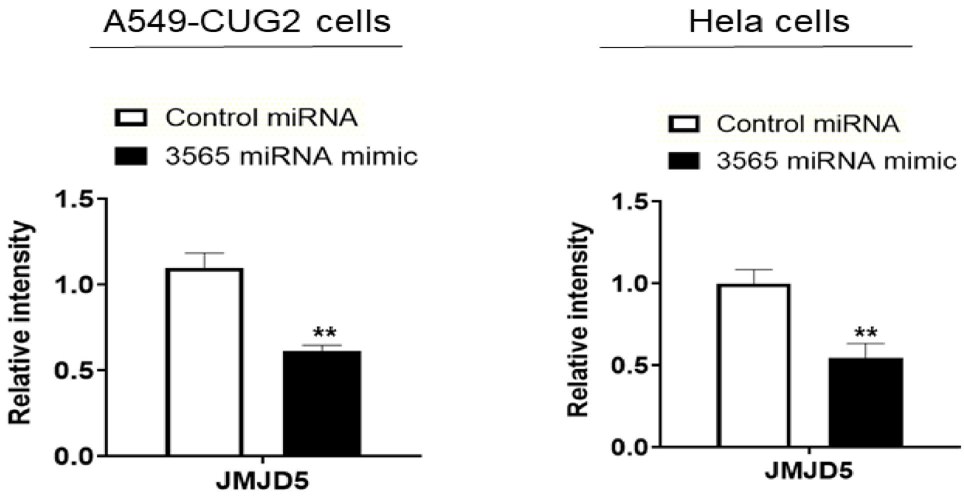

Decreased levels of *JMJD5* transcripts after treatment with the miR-3656 mimic

A real-time RT-PCR was conducted on an AriaMX Real-time system (Agilent Technologies, Santa Clara, USA) using the SYBR Premix EX Taq (Takara, Shiga, Japan) and the following gene-specific primers: *JMJD5,* (forward) 5’-GGAACCATCTCCCCACTACAT-3’ and (reverse) 5’-CTGGCTCGTGTTATGGAGAAG-3’, *GAPDH* (forward) 5’-CTTTGGTATC GTGGAAGGACTC - 3’ and (reverse) 5’-GTAGAGGCAGGGATGATGTTCT -3’. Real-time RT-PCR data were obtained in the form of threshold cycle (Cq) values, and target gene

expression was normalized to GAPDH expression. Relative expression levels of *JMJD5* were calculated using the comparative Cq ($2^{-\triangle\triangle Cq}$) method. The assay was conducted in triplicate. The data designate the mean $\pm$ SD. (** p<0.01, miR-3565 *vs* miR-control)

Supplementary Figure S3

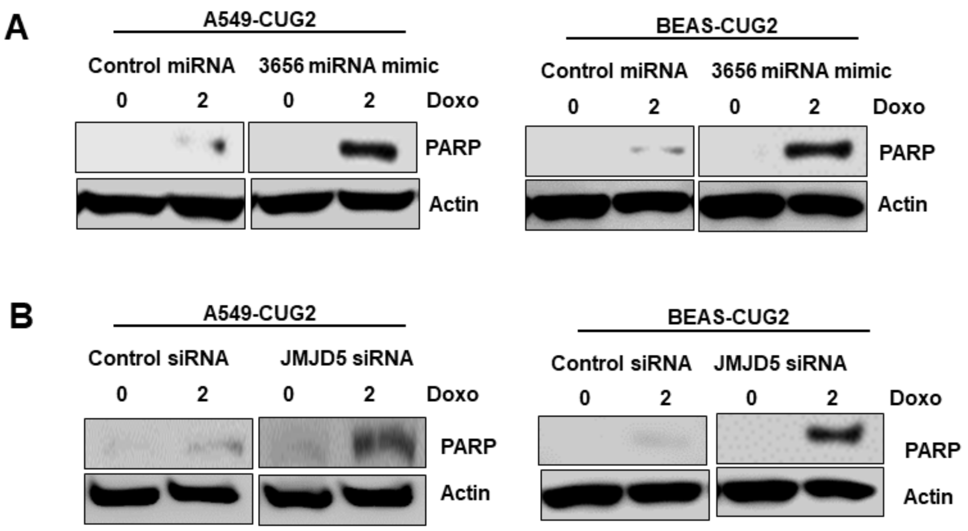

Sensitization of apoptosis after treatment with the miR-3656 mimic or JMJD5 siRNA under doxorubicin

Under doxorubicin (2 μg/ml), A549-CUG2 and BEAS-CU2 cells were treated with the miR-3656 mimic or JMJD5 siRNA. Cleaved PARP was detected for apoptosis 2 days post-transfection using immunoblotting.

Supplementary Figure S4

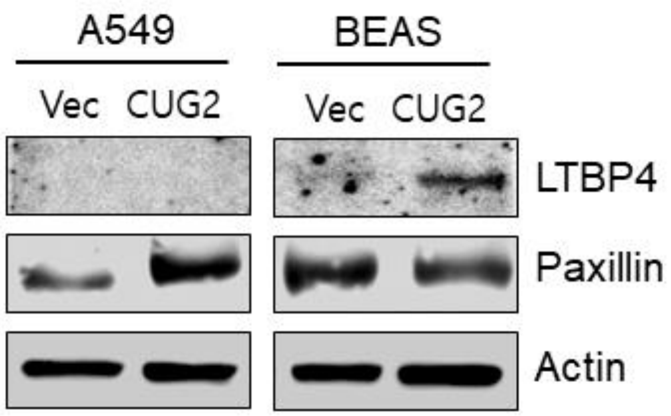

Other candidates of target proteins against miR-3656

LTBP4 and paxillin protein levels in A549-CUG2 and BEAS-CUG2 cells were compared with their control cells with immunoblotting.

Supplementary Figure S5

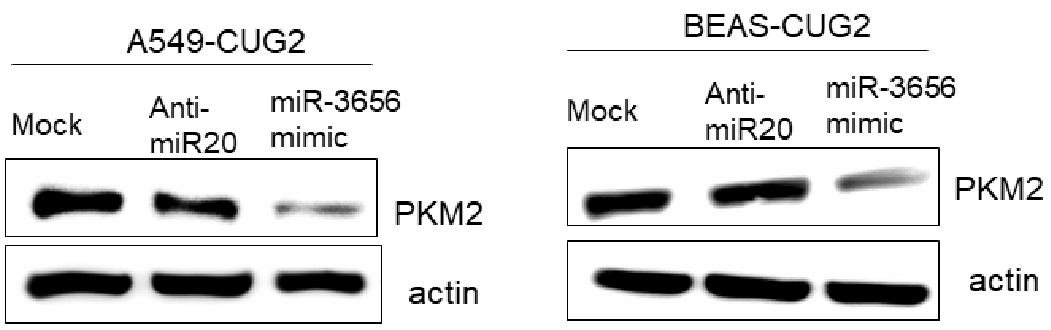

Decreased levels of PKM2 protein after treatment with the miR-3656 mimic

A549-CUG2 and BEAS-CU2 cells were treated with the miR-3656 mimic or anti-miR20. PKM2 protein was detected 2 days post-transfection using immunoblotting.
